# Assessment and strategy development for SARS-CoV-2 screening in wildlife: A review

**DOI:** 10.14202/vetworld.2023.1193-1200

**Published:** 2023-06-04

**Authors:** Jignesh Italiya, Tanvi Bhavsar, Jiří Černý

**Affiliations:** 1 Centre for Infectious Animal Diseases, Faculty of Tropical Agrisciences, Czech University of Life Sciences Prague, Kamýcká 129, 165 00 Prague – Suchdol, Czechia; 2 Animal Physiology Division, ICAR-National Dairy Research Institute, Karnal, Haryana, India

**Keywords:** angiotensin-converting enzyme 2, coronavirus disease 2019 in animals, severe acute respiratory syndrome coronavirus-2, wildlife surveillance

## Abstract

Coronaviruses (members of the *Coronaviridae* family) are prominent in veterinary medicine, with several known infectious agents commonly reported. In contrast, human medicine has disregarded coronaviruses for an extended period. Within the past two decades, coronaviruses have caused three major outbreaks. One such outbreak was the coronavirus disease 2019 (COVID-19) caused by the coronavirus severe acute respiratory syndrome coronavirus-2 (SARS-CoV-2). Over the 3-year COVID-19 outbreak, several instances of zooanthroponosis have been documented, which pose risks for virus modifications and possible re-emergence of the virus into the human population, causing a new epidemic and possible threats for vaccination or treatment failure. Therefore, widespread screening of animals is an essential technique for mitigating future risks and repercussions. However, mass detection of SARS-CoV-2 in wild animals might be challenging. *In silico* prediction modeling, experimental studies conducted on various animal species, and natural infection episodes recorded in various species might provide information on the potential threats to wildlife. They may be useful for diagnostic and mass screening purposes. In this review, the possible methods of wildlife screening, based on experimental data and environmental elements that might play a crucial role in its effective implementation, are reviewed.

## Introduction

With the increasing human population, climate change, and human interference in wildlife ecosystems over the past few decades, many emerging infectious diseases (EIDs) have developed. The ongoing coronavirus disease 2019 (COVID-19) pandemic is one of them. The novel zoonotic coronavirus, namely, severe acute respiratory syndrome coronavirus-2 (SARS-CoV-2), belongs to the order Nidovirales, suborder Cornidovirineae, family *Coronaviridae*, subfamily Orthocoronavirinae, genus *Betacoronavirus*, and subgenus *Sarbecovirus* [1]. The causative agent of the ongoing pandemic in humans has also demonstrated the ability to infect different animal species [2].

Over the past two decades, three major epidemic and pandemic outbreaks have been reported due to viruses from this family, especially from the *Betacoronavirus* genus [3]. The first epidemic of SARS-CoV-1 was reported in Foshan, Guangdong, China, in 2001. Horseshoe bats, from the genus *Rhinolophus* and palm civets have been identified as natural reservoirs for SARS-CoV-1 [4]. In 2012, a second outbreak in the Middle East was reported, caused by the Middle East respiratory syndrome coronavirus (MERS-CoV). According to the latest WHO report, it resulted in 2519 cases with 866 deaths [5]. Dromedary camels were identified as reservoirs for MERS-CoV [6]. In addition, one outbreak on pig farms was reported; swine enteric alphacoronavirus, or swine acute diarrhea syndrome coronavirus (SADS-CoV), was discovered in pig farms in Guangdong province, China, in 2017. It initially appeared as outbreaks of severe diarrhea in suckling piglets within four swine herds in a mountainous area of northern Guangdong [7]. Later, it reemerged in pig herds in Guangdong, starting in February 2019, and caused the mortality of about 2000 pigs [8]. Swine acute diarrhea syndrome coronavirus originated in bats, like other zoonotic viruses, including SARS-CoV and MERS-CoV [9].

Humans, domestic animals, wildlife, and the environment are linked by their different roles in transmitting and maintaining infectious agents [10]. Recent coronavirus outbreaks have increased the focus on disease surveillance and identification of other pathogenic organisms in wild animals. Wildlife disease surveillance will bring benefits to conservation efforts and the monitoring, prevention, and control of zoonotic diseases. Increased wildlife disease surveillance and disease ecology modeling data were generated through the widespread application of molecular tools to expand the knowledge of different infectious agents and possible future EIDs. The concept of wildlife disease surveillance is similar to domestic animal health surveillance. However, the ecological and behavioral characteristics of wildlife populations and some significant differences compared with domestic animal populations must be considered when planning and implementing wildlife health surveillance projects [11].

In this review, the available information on SARS-CoV-2 in wild animals was analyzed, as well as its implementation in planning and preparing wildlife health surveillance efforts and specific pathogen surveillance.

## Risk Assessment of SARS-CoV-2 Exposure in Free-ranging Wild Animals

Risk assessment of wildlife health includes assessing the hazard release from the source, the hazard exposure, and its consequences [12].

### Source of SARS-CoV-2

Infectious SARS-CoV-2 is present in the respiratory secretions of infected humans, pet animals, captive wild animals, and production animals (e.g., minks). Humans could be a potential source of infection for free-ranging wild animals due to the high infection rates of SARS-CoV-2 in humans [13]. Severe acute respiratory syndrome coronavirus-2 was also discovered in the feces and urine of infected human patients [14, 15]. It has been observed that SARS-CoV-2 can survive on non-living substances such as plastic waste and masks. For instance, SARS-CoV-2 can survive for 21 days on plastic, 14 days on stainless steel, 7 days on nitrile gloves, and 4 days on chemical-resistant gloves [16]. A recent study reported multiple spillovers from humans and onward transmission of SARS-CoV-2 in white-tailed deer, which highlights an urgent need for a robust and responsive “One Health” approach to obtain an enhanced understanding of the ecology, molecular evolution, and dissemination of SARS-CoV-2 [17].

### Exposure to SARS-CoV-2

The transmission of SARS-CoV-2 primarily occurs through respiratory droplets and airborne aerosols [18]. When in close contact with humans, cases of animal infection have been reported among pet animals and zoo-kept wildlife [13]. Human waste can be the source of infections for wild animals, and free-living animals in the human population could be the potential linkage between humans and wild animals for SARS-CoV-2 infection. Handling, keeping, caring for, and releasing wild animals may expose them to diseases transmitted by infected handlers. Biologists, wildlife veterinarians, forest workers, and people living near protected areas could be the source of animal infections.

### Consequences of SARS-CoV-2 infection

The occurrence of SARS-CoV-2 infections in wild animals has an impact on animal, as well as human health. Severe acute respiratory syndrome coronavirus-2 diseases in wild animals impact the welfare and conservation of wild animals [19, 20]. In addition, it also affects virus mutation once it crosses the species barrier [21]. Such mutations have been observed in mink infection cases [22]. Several cases have been reported worldwide of SARS-CoV-2 transfer from humans to minks. During the natural passage of this virus in minks, several mutations have been observed, mostly in spike protein S, the most important SARS-CoV-2 structural protein. These include Y453F, F486L, and N501T [23]. N501T has shown a greater ability to bind to mink angiotensin-converting enzyme 2 (ACE2), the SARS-CoV-2 receptor, and therefore leads to more effective use of mink ACE2 receptors for SARS-CoV-2 entrance [24]. According Porter *et al*. [25], the mutation Y486F occurred early in various mink outbreaks, and the mutations F486L and Q314K may co-occur. This demonstrates that SARS-CoV-2 experiences a transient, but significant, increase in evolutionary pace in response to increased selection pressures during species jumps, which may result in mink-specific mutations [25]. A recent study revealed the existence of five mutation sites typical of all early human-isolated SARS-CoV-2 Omicron variants. These mutations adapted the virus to infect mice, indicating that Omicron may have evolved in a mouse host [26].

## Role of Surveillance in the Investigation of EIDs

The majority of EIDs originate from wildlife; they pose a zoonotic threat and often have a considerable impact on society [27]. To avoid future zoonotic outbreaks, it is essential to maintain the integrity of ecosystems and other crucial measures, such as critical measures on wildlife trade and building proper surveillance systems around this trade. Monitoring and surveillance are important to the understanding of emerging epidemiological situations. They should be used in response to disease threats and outbreaks and when considering the risk of wild animal translocations. In the context of animal health, wildlife disease surveillance provides information about disease patterns, epidemiology, and intensity, identifies changes in patterns of disease occurrence over time, and assists in the early detection of potential outbreaks, according to the World Organization for Animal Health [28].

Over the past two decades, the growing frequency of outbreaks from the *Coronaviridae* family has increased pathogen-specific surveillance, which has resulted in the identification of some new viruses with zoonotic potential. The implication that bats could act as possible progenitors of emerging coronaviruses prompted global surveillance activities and resulted in the identification of different bat coronaviruses from other bat species with cross-species transmission events [29]. Moreover, after the SARS-CoV-1 outbreak, several animal coronaviruses related to HCoV229E, HCoVNL63, MERS-CoV, and SARS-CoV were found in different African countries [30].

Similarly, a 5-year surveillance program (from 2011 to 2015) carried out in a single cave inhabited by multiple species of horseshoe bats in Yunnan Province, China, revealed 15 severe acute respiratory syndrome-related coronavirus strains (11 novel ones and four that are known from the previous studies) [31].

## Different Surveillance Strategies and Their Implementation in the Current Pandemic

The World Organization for Animal Health defined surveillance in an epidemiological sense as the ongoing recording of disease in animal populations from the disease management perspective [11]. The first step of any disease surveillance program is to identify the goal(s). Once the system is established, it may vary depending on the desired outcome. Surveillance output can include the detection of new diseases, declaring a population free of specific diseases or infections, or identifying disease levels and distributions in the population [32].

Surveillance is mainly divided into two categories: active surveillance and passive surveillance. Active surveillance includes actively searching for particular diseases, while passive surveillance involves continuously searching for diseases on an *ad hoc* basis [33]. Passive surveillance includes vector surveillance, sentinel surveillance, serological surveillance, pathogen surveillance, and participatory surveillance. In comparison, active surveillance includes clinical investigation, syndromic surveillance, mortality investigation, and parameter monitoring [34]. Among these different surveillance modalities, described in Table-1, some have been found to be valuable for the current SARS-CoV-2 pandemic. During the current pandemic situation, pathogen detection, or target surveillance, and serological surveillance are commonly implemented.

**Table-1:** Different surveillance modalities that can be useful for the current pandemic.

S. No.	Specific category	Description
1.	Pathogen determination	Search for a specific pathogen (or its antigens or nucleic acids)
2.	Serological determination	Search for antibodies against a specific pathogen
3.	Clinical investigation	Monitoring the clinical signs compatible with the disease (s)
4.	Parameter monitoring	Screening of biological indicators (e.g., food intake, fecal output, body weight, and animal activity)

Clinical investigation can be conducted by observing clinical signs reported in natural infection cases and experimental infection demonstrations. Several clinical signs have been observed in different animals infected with SARS-CoV-2, summarized in Table-2 [28, 35–47].

**Table-2:** Common clinical signs observed in different species with SARS-CoV-2 infection based on data from the world organization for animal health.

Animal species	Observed clinical signs	Reference
Cat	Anorexia, sneezing, acute dyspnea, rattle, snoring, nasal secretion, severe respiratory failure, lethargy, breathing difficulties, and digestive signs	[28, 35]
Dog	Conjunctivitis, cough, rhinitis, dyspnea and weakening, high respiratory distress and apathy, nasal discharge and fever, febrile peaks, anorexia, abnormal lung sounds, pharyngitis, bronchitis, lymphadenomegaly, and positive palmopercussion	[36, 37]
Mink	Respiratory symptoms, high mortality & anorexia	[38]
Lion	Mild-to-moderate symptoms in the upper respiratory tract (serous nasal discharge, sneezing, and coughing	[28, 39]
Puma	Anorexia	[40]
Hyenas	Extremely mild symptoms, including slight lethargy, some nasal discharge, and occasional coughs	[28]
Ferret	Clinical signs of gastrointestinal tract	[41]
Snow leopard	Coughing and some wheezing	[42]
Gorilla	Tiredness, dry cough, and loss of appetite	[43]
Amur leopard cat	Serous and bloody nasal discharge and rhinitis	[28, 44]
Malayan tigers	Growl and wheeze, followed by coughing, nasal discharge, lethargy, and loss of appetite	[28, 45]
Sumatran tiger	Growl and wheeze, followed by coughing, nasal discharge, lethargy, and loss of appetite	[28, 46]
Hippopotamus	Mild symptom like nasal discharge	[47]

## Fundamental Challenges and Strategy Development for SARS-CoV-2 Mass Screening in Wild Animals

Mass screening could be implemented using different surveillance modalities such as pathogen determination, serological determination, clinical investigation, and parameter monitoring. However, with current pandemic situations and considerations, target pathogen detection and serological surveillance could be essential tools to use. For example, Jemeršić *et al*. [48] conducted serological surveillance and pathogen detection in free-living and captive animals during the first wave of COVID-19 in Croatia.

**Figure-1:**
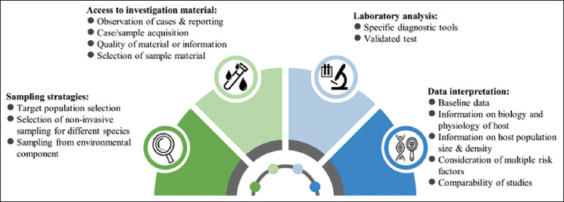
Fundamental challenges of severe acute respiratory syndrome coronavirus-2 screening in wildlife.

The mass screening of wild animals for SARS-CoV-2 is quite challenging regarding budget, planning, preparation, and implementation of the strategy, and meeting the desired goals. In general, there are several challenges listed for wildlife surveillance. The unique challenges regarding wildlife disease surveillance are the detection of disease and pathogens in these animals. In wild animals, the signs of illness are often not obvious when diseased, especially subclinical infections, and observation and/or access to dead animals are difficult due to the rapid removal by predators and scavengers [49]. In addition, the cost implications are also a big challenge for surveillance programs. Thus, it is important to regularly evaluate large-scale active surveillance programs to ensure that goals are being met. Figure-1 depicts the fundamental challenges of SARS-CoV-2 mass screening in wildlife, including sampling strategies, access to the investigatory material, laboratory analysis, and data interpretation.

### Sampling strategies

During targeted surveillance or pathogen-specific surveillance, studies are conducted in which statistical inferences about the population of interest are very limited [50]. This is usually caused by many factors, for example, limited numbers of sampled individuals since most of the sampling is opportunistic and large sampling campaigns can be too expensive to perform. Then, sampling can be very complicated or impossible due to either laws and regulations or practical issues, as these animals can be too difficult to trap and handle. During targeted surveillance studies, a cohort of the population of interest is targeted based on a high-risk for exposure and susceptibility rates [11]. These studies may focus on populations of animals that seem to be in good health conditions [50]. Regarding SARS-CoV-2 virus detection in wild animals, target species populations can be divided into three groups based on previous known natural infection events, experimental studies, and *in silico* studies: high-risk susceptibility (or first target group), medium-risk (or second target group), and low-risk (or third target group).

The viral spike proteins (S) are the primary determinant of the host cell [51]. During host cell entry, they play a key role in the attachment process to the host cell-surface receptor, ACE2 protein [52]. There are several mammalian species that conserve these protein sequences. Based on the presence of ACE2 receptors, predicting the permissive animal species for natural infection with SARS-CoV-2 is possible. The transmembrane serine protease-2 also plays a key role in the attachment of the virus to the host cell [53]. However, *in silico* studies are limited to host cell entrance, and replication may also depend on numerous other variables, such as proteases Cathepsin L) and a disintegrin and metalloprotease domain [54]. The expression of ACE2 proteins in different species not only indicates the possibilities of natural infection but also shows host entry, the involvement of different tissue types, and the clinical expression of the disease, which were revealed by studies with COVID-19 human patients [55]. Based on these bioinformatic studies, Alexander *et al*. [56] identified five animal species that are highly susceptible to SARS-CoV-2 infections, including the Rhesus macaque, house cat, tiger, lion, and golden Syrian hamster.

Since the beginning of the pandemic, several animal species have been found to be susceptible to infection, which supports the *in silico* findings. For instance, the exposure of SARS-CoV-2 in white-tailed deer was demonstrated by serosurveillance [57], which supported the *in silico* modeling data [58]. Therefore, based on the high-risk susceptibility of these animals, as shown through *in silico* findings, experimental infection results, and some natural infection cases, animals such as white-tailed deer could be the first target animal population for pathogen-specific surveillance or serosurveillance. On the other hand, animal species that are identified as high-risk regarding susceptibility based on *in silico* findings, but no natural infection events or experimental infection cases are recorded yet, fall under the second target animal population.

### Access to investigation material

Sampling methods are primarily selected based on the chosen surveillance modalities. It also includes a stratified random sampling of the population of interest. During stratified random sampling, a subunit of the population is sampled based on known risk factors [59]. A sample can be collected opportunistically during routine operations, or animals can be handled and captured for sampling purposes. Among the invasive and non-invasive methods of sampling, non-invasive sampling methods are always preferred in wildlife surveillance [60].

**Figure-2:**
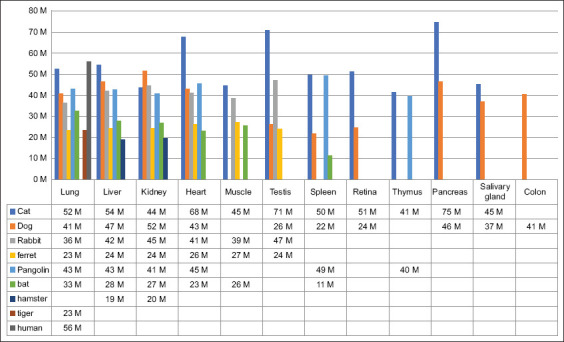
Expression of the angiotensin-converting enzyme 2 gene in different tissues of different species (original transcripts per kilobase of exon model per million mapped reads). The bar graph was prepared based on data from [21].

Sample selection for surveillance also depends on the chosen analysis strategy and targeted virus tissue tropism in different animal species. Depending on the expression of ACE2 receptors in different tissues of different animals, the susceptibility of infection and its clinical manifestation varies [58]. Based on that, the clinical outcome of the disease and sampling strategies can be determined. For example, SARS-CoV-2 was detected in rectal swabs from infected ferrets and dogs [61]. Thus, non-invasive samples were also selected as investigatory materials based on tissue tropism and experimental studies. In Figure-2 [21], the expression of the ACE2 gene in different tissues of different species has been demonstrated. Aguiló-Gisbert *et al*. [19] detected SARS-CoV-2 in 2 of 13 feral dark brown American minks (*Neovison vison*) trapped in the Valencian community (Eastern Spain) during an invasive species trapping campaign. The virus was found in mesenteric lymph nodes of animals. Sampling dead animals could also be an option; however, scavengers can remove them rapidly, as mentioned.

In terms of environmental sampling, it is critical to collect samples from common water sources for wildlife as well as from human waste in the local ecosystem because it has been discovered that infected human waste can contaminate the local ecosystem and serve as a source of infection [20].

### Laboratory analysis and data interpretation

Since the beginning of the pandemic, several diagnostic tests have been developed. The diagnostic assay includes virus culture, nucleic acid testing assays, and immunological assays. Real-time reverse transcription-quantitative polymerase chain reaction (RT-qPCR) is one of the best methods for detecting SARS-CoV-2 RNA [62]. However, loop-mediated isothermal amplification could serve as an alternative method to RT-qPCR to detect SARS-CoV-2 RNA. This method can be used without the need for specialized equipment and trained analysts [63].

There has also been an immunological assay enzyme-linked immunosorbent assay (ELISA) methodology developed to diagnose the presence of antibodies against SARS-CoV-2 in animals. For example, Wernike *et al*. [64] developed an indirect multispecies ELISA based on the receptor-binding domain for ferrets, raccoon dogs, hamsters, rabbits, chickens, cattle, and cats. Serological surveillance (using a commercial ELISA kit) revealed the presence of antibodies against SARS-CoV-2 in sheep and goats, confirmed by a virus neutralization test [65].

Data interpretation plays a crucial role in the development and validation of an assay. For serological assays, cross-immunity against similar virus antigens is a major drawback. Following virus nucleic acid detection, it is critical to perform sequencing to identify novel changes or mutations in the virus genome to overcome its future consequences. Further, actions should be taken based on the achieved results, For example, several mass culling of minks were carried out after the identification of infection spillover and mutation [66].

## Conclusion

To develop strategies and identify challenges for SARS-CoV-2 screening, the current knowledge of SARS-CoV-2 infection in animals plays a significant role. Continued assessment of the risk of SARS-CoV-2 infection in animals aids in breaking the link between virus exposure and wild-living animals. Natural infection cases reported in different zoos worldwide provide baseline data on the severity of infections and virus biology in wild animals. Collective data from various sources, such as *in silico* studies, experimental infection case studies, and natural infection, aid in developing mass wildlife screening strategies and resolving challenges.

In the future, continued upgrading of knowledge and identifying new animal hosts susceptible to SARS-CoV-2 infection during the current pandemic will help modify disease surveillance strategies in wildlife.

## Authors’ Contributions

JI: Conceptualization, writing original draft, and visualization. JI and JC: Writing-review and editing. JI and TB: Resources. JC: Supervision. All authors have read, reviewed, and approved the final manuscript.
